# Losartan controls immune checkpoint blocker-induced edema and improves survival in glioblastoma mouse models

**DOI:** 10.1073/pnas.2219199120

**Published:** 2023-02-01

**Authors:** Meenal Datta, Sampurna Chatterjee, Elizabeth M. Perez, Simon Gritsch, Sylvie Roberge, Mark Duquette, Ivy X. Chen, Kamila Naxerova, Ashwin S. Kumar, Mitrajit Ghosh, Kyrre E. Emblem, Mei R. Ng, William W. Ho, Pragya Kumar, Shanmugarajan Krishnan, Xinyue Dong, Maria C. Speranza, Martha R. Neagu, J. Bryan Iorgulescu, Raymond Y. Huang, Gilbert Youssef, David A. Reardon, Arlene H. Sharpe, Gordon J. Freeman, Mario L. Suvà, Lei Xu, Rakesh K. Jain

**Affiliations:** ^a^Edwin L. Steele Laboratories, Department of Radiation Oncology, Massachusetts General Hospital and Harvard Medical School, Boston, MA 02114; ^b^Department of Pathology and Center for Cancer Research, Massachusetts General Hospital, Harvard Medical School, Boston, MA 02114; ^c^Broad Institute of MIT and Harvard, Cambridge, MA 02142; ^d^Department of Systems Biology, Harvard Medical School, Boston, MA 02115; ^e^Harvard-Massachusetts Institute of Technology Division of Health Sciences and Technology, Massachusetts Institute of Technology, Cambridge, MA 02142; ^f^Department of Physics and Computational Radiology, Division of Radiology and Nuclear Medicine, Oslo University Hospital, Oslo, 0372 Norway; ^g^Department of Chemical Engineering, Massachusetts Institute of Technology, Cambridge, MA 02142; ^h^Department of Medical Oncology, Dana-Farber Cancer Institute, Boston, MA 02115; ^i^Department of Medicine, Harvard Medical School, Boston, MA 02115; ^j^Department of Immunology, Blavatnik Institute, Harvard Medical School, Boston, MA; ^k^Department of Radiology, Brigham and Women’s Hospital, Boston, MA 02115; ^l^Center for Neuro-Oncology, Dana-Farber Cancer Institute, Boston, MA 02215

**Keywords:** immune checkpoint blockers, glioblastoma, immune-related adverse events, tumor microenvironment, biomarkers

## Abstract

Immune checkpoint blockers (ICBs) have failed in all phase III glioblastoma trials. Here, we found that ICBs induce cerebral edema in some patients and mice with glioblastoma. Through single-cell RNA sequencing, intravital imaging, and CD8^+^ T cell blocking studies in mice, we demonstrated that this edema results from an inflammatory response following antiprogrammed death 1 (PD1) antibody treatment that disrupts the blood–tumor barrier. Used in lieu of immunosuppressive corticosteroids, the angiotensin receptor blocker losartan prevented this ICB-induced edema and reprogrammed the tumor microenvironment, curing 20% of mice which increased to 40% in combination with standard of care treatment. Using a bihemispheric tumor model, we identified a “hot” tumor immune signature prior to losartan+anti-PD1 therapy that predicted long-term survival. Our findings provide the rationale and associated biomarkers to test losartan with ICBs in glioblastoma patients.

## Significance

Improving immunotherapy outcomes for the majority of glioblastoma patients remains a critically unmet need. In mouse models of glioblastoma, the use of a safe, affordable, and widely prescribed antihypertensive agent (losartan) overcomes immune-related adverse events, enhances antitumor immune activity, and improves survival outcomes of immune checkpoint blocker therapy. A mouse biomarker model provides key insights into cellular mediators of immunotherapy response that are present in the tumor microenvironment prior to treatment. The results shown here serve as a foundation for future clinical studies testing the combination of losartan with immune checkpoint blockade in glioblastoma patients.

Despite reports that some murine glioblastoma (GBM) models can be cured with immune checkpoint blockers (ICBs), this immunotherapeutic approach has failed in all phase III GBM clinical trials. A challenge unique to GBM is the cerebral edema which can be exacerbated by antiprogrammed death/ligand 1 (PD1/PD-L1) antibodies (1, 2). Currently, this increased edema is controlled by potent, immunosuppressive steroids that compromise ICB efficacy.

Here, we demonstrate that the angiotensin receptor blocker (ARB) losartan prevents ICB-induced edema by reducing tumor endothelial cell (TEC) expression of membrane-type matrix metalloproteinases 1 and 2 (MT-MMP-1, -2) that are upregulated during treatment with an anti-PD1 antibody. Furthermore, losartan increases GBM perfusion, enhances anti-tumor immunity, and improves survival (in two out of three models) under anti-PD1 treatment. Utilizing a bihemispheric model, we show that immune composition in the tumor microenvironment (TME) prior to treatment predicts individual and differential responses.

## Results

### ICB Treatment Disrupts the GBM Vasculature and Induces Edema.

**Fig. 1.**
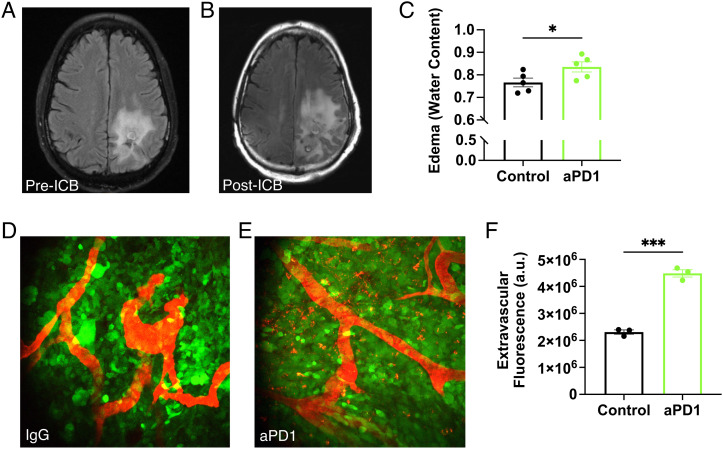
ICB increases GBM vascular leakage and induces brain edema. MR T2-weighted-Fluid-Attenuated Inversion Recovery (T2-FLAIR) images obtained from a recurrent GBM patient (*A*) before and (*B*) after 4 mo of anti-PD-L1 (MEDI4763; NCT02336165) treatment show increased edema after ICB treatment. In addition to ICB-induced inflammation, this change may be due in part to underlying tumor activity or growth. (*C*) In mice, anti-PD1 antibody (aPD1) treatment increases edema in GL261 tumors compared to IgG control [as measured by wet-dry weight (i.e., water content) evaluation of tumor tissue; n = 5]. Multiphoton visualization of the brain vasculature via injected tetramethylrhodamine (TAMRA) labeled albumin (red) imaged through transparent cranial windows in mice bearing GFP+ GL261 GBM (green) shows that compared to IgG controls (*D*) there is increased extravasation in anti-PD1-treated tumors after the third consecutive dose (*E*). (*F*) Quantification shows that more albumin in anti-PD1-treated mice has leaked outside of the tumor blood vessels (n = 3). (Bar plots: mean ± SEM; Student’s unpaired *t* test; *P < 0.05; ***P < 0.001.)

MRI revealed ICB-induced edema in some GBM patients (Fig. 1 *A* and *B*). We analyzed our institutional patient cohort of ICB-treated GBM patients to determine the percent increase in the extent of peritumoral edema in the first 6 mo post therapy (*SI Appendix*, Table S1). We found that the median percentage increase in edema was 18.8% (−29.6 to 123.5% interquartile range). Factors associated with edema increase included baseline edema volume prior to treatment and radiotherapy treatment; bevacizumab was associated with a decrease in edema. In multivariable Cox regression analysis, neither the patient’s baseline edema volume nor their maximum change in edema within 6 mo of starting of ICB was associated with overall survival (OS) as measured from the start of ICB treatment (*SI Appendix*, Table S2).

In the GL261 model, anti-PD1 antibody treatment recapitulated this increased edema (Fig. 1*C*). We performed intravital microscopy after injecting the mice with a fluorescent tracer to detect vascular leakage. We found that tumor vessels in control (IgG-treated) mice retained most of the tracer (Fig. 1*D*), but in anti-PD1-treated mice (Fig. 1*E*), excess tracer leaked into the surrounding tissue (Fig. 1*F*), indicating endothelial barrier disruption. Because losartan and other ARBs have been shown to lower vascular endothelial growth factor (VEGF) expression in GBM models and vasogenic edema in retrospective patient studies (3–5), we decided to test the effects of losartan treatment on ICB-induced edema.

### Losartan Prevents ICB-Induced Edema by Reducing TEC MT-MMP-1 and -2 Expression.

**Fig. 2.**
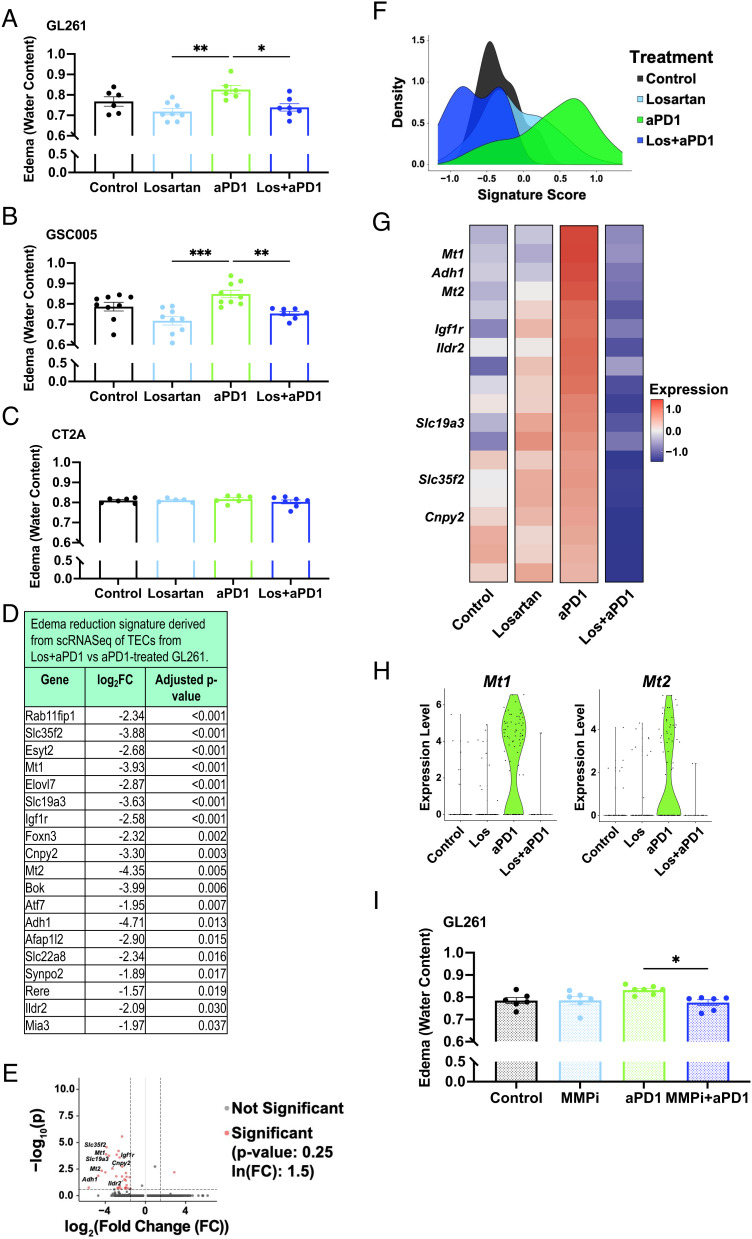
Losartan prevents ICB-induced edema by downregulating TEC MT-MMP-1 and -2 expression. Losartan decreases anti-PD1-induced edema in (*A*) GL261 and (*B*) 005 GSC models but not in (*C*) CT2A after 2 wk of treatment (n = 5 to 9). (*D*) scRNASeq of TECs reveals a set of downregulated genes that includes those related to metabolism (e.g., *Adh1, Ildr2*), angiogenesis/migration (e.g., *Cnpy2, Igf1r*), and solute carriers (e.g., *Slc35f2, Slc19a3*). When applied as an edema signature, this gene set is upregulated in anti-PD1-treated GL261 tumors compared to other treatment arms as visualized via (*E*) volcano plot, (*F*) density plot of edema signature scores (methods described in *SI Appendix*) by treatment and (*G*) mean gene expression heat map of edema signature genes. (*H*) Specialized MT-MMPs (*Mt1, Mt2*) are among these genes and are expressed in TECs only from the anti-PD1-treated tumors. (*I*) The MMP inhibitor Ilomastat (MMPi) controls anti-PD1-induced edema comparably to losartan in GL261 (n = 6). (Edema signaturegene expression units = ln(TP100k + 1); log_2_FC = fold changes > |2|; adjusted *P* value < 0.05. Bar plots: mean ± SEM; one-way ANOVA with Tukey’s post hoc test; *P < 0.05; **P < 0.01; ***P < 0.001.)

In the GL261 and 005 GSC (glioma stem cell) models (Fig. 2 *A* and *B*), but not in CT2A (Fig. 2*C*), we found that anti-PD1 treatment increased edema, while losartan prevented this anti-PD1-induced edema. To reveal the edema-reduction mechanism, we performed single-cell RNA sequencing (scRNASeq) on TECs in the GL261 model (*SI Appendix*, Fig. S1 and Dataset S1). We identified a set of genes downregulated in TECs from losartan+anti-PD1-treated tumors vs. anti-PD1 monotherapy (Fig. 2 *D* and *E* and Dataset S2). This edema signature was most highly expressed in TECs from anti-PD1-treated tumors (Fig. 2 *F* and *G*). Genes included those related to metabolism, angiogenesis/migration, solute carriers, and most notably, a specific subset of MT-MMPs (*Mt1* and *Mt2*, i.e., MMP-14 and MMP-15). Interestingly, we did not observe gene expression changes in VEGF/VEGFRs or other known vasogenic edema-related genes in this TEC signature (Fig. 2 *D* and *E*). Thus, we explored possible inflammatory mechanisms governing ICB-induced edema.

We found via scRNASeq (*SI Appendix*, Fig. S2 and Dataset S3) and T cell blocking experiments (*SI Appendix*, Fig. S3) that CD8^+^ T cells are important mediators of ICB-induced edema. Because MMP overexpression in endothelial cells has been linked to blood–brain barrier (BBB) tight junction disruption and cerebral edema (6, 7) and can be induced by CD8^+^ T cell interactions (8), we hypothesized that this could be a potential mechanism of ICB-induced edema in GBM. Indeed, *Mt1* and *Mt2* are only expressed in TECs from anti-PD1-treated tumors (Fig. 2*H*). To test this mechanism, we gave Ilomastat, a broad-spectrum MMP inhibitor that is nontoxic to GBM cells at physiological levels (9), to mice bearing GL261 tumors under anti-PD1 treatment. We found that Ilomastat phenocopied the ability of losartan to prevent anti-PD1-induced edema (Fig. 2*I*). Because ARBs can modulate other TME features (10–13), we next evaluated the effects of losartan on GBM extracellular matrix (ECM), vasculature, and immune components.

### Losartan Reduces ECM and Solid Stress, Normalizes the Tumor Vasculature, Improves Perfusion, and Decreases Hypoxia and Immunosuppression in GBM.

**Fig. 3.**
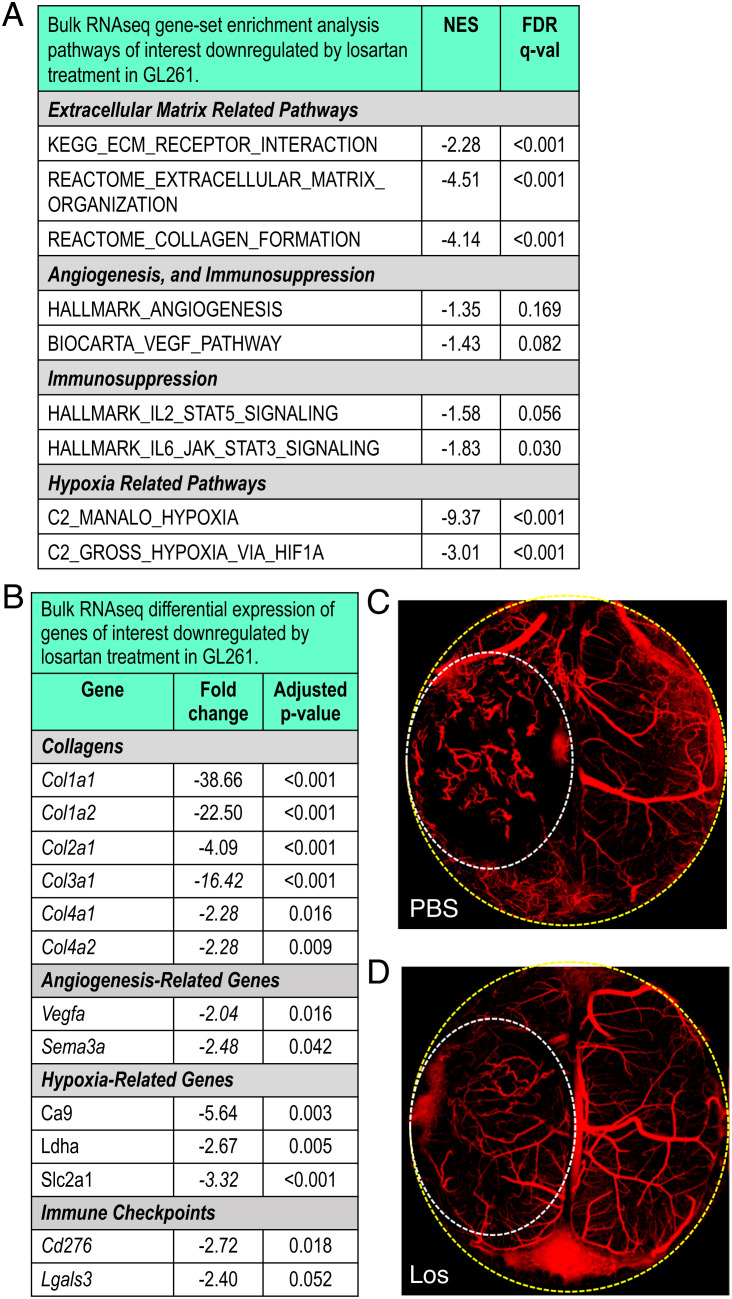
Losartan reprograms the GBM tumor microenvironment. (*A*) TME-related gene-set enrichment analysis pathways downregulated by losartan treatment compared to control in bulk RNASeq of GL261 tumors (n = 3). (*B*) Differential gene expression confirms these effects in matrix molecules such as collagen, hypoxia-related genes, and immune checkpoints. Intravital OCT imaging [to detect perfused vessels (red) vs. nonperfused areas (black)] shows that compared to PBS-treated controls (*C*), losartan (*D*) renders tumor blood vessels less tortuous and improves tumor perfusion (yellow dashed line—cranial window border; white dashed line—tumor area). (Sequencing: FDR, false discovery rate; all FDR q-values < 0.20; NES, normalized enrichment score; all adjusted *P* values < 0.05, FC > |2|. Bar plots: mean ± SEM; Student’s unpaired *t* test; *P < 0.05.)

Losartan lowers collagen and hyaluronic acid (HA) levels in extracranial tumors, reducing the compressive “solid stress,” thereby decompressing previously collapsed blood vessels (11). Using bulk RNASeq in GL261, we found that losartan treatment significantly reduced gene expression related to ECM, angiogenesis, immunosuppression, and hypoxia compared to controls (Fig. 3 *A* and *B*). We observed reduced expression of immune checkpoints both at the transcriptional (Fig. 3*B*) and protein (*SI Appendix*, Fig. S4) levels. Because HA is a major GBM ECM component, we confirmed via immunohistochemistry that losartan lowers HA levels (*SI Appendix*, Fig. S4). To test if this reduced solid stress, we analyzed tumor tissue deformation [i.e., a measure of solid stress (14)] and found a reduction in losartan-treated tumors (*SI Appendix*, Fig. S4). We found that losartan did not exert cytotoxic nor any other significant effects on the malignant cell population via scRNASeq (*SI Appendix*, Fig. S5).

We next determined whether losartan improved vascular function in GBM. Using optical coherence tomography (OCT) (15), we found that control tumors featured chaotic abnormal vessels and nonperfused regions (Fig. 3*C* and Movie S1), whereas losartan-treated tumors had more normalized, straighter, decompressed vessels with greater overall perfusion (Fig. 3*D* and Movie S2). In perfusion-MR images, we found that GBM patients receiving losartan or other angiotensin system inhibitors (ASIs) also had improved tumor perfusion (*SI Appendix*, Fig. S6).

### Losartan Repolarizes Myeloid Cells from Pro- to Antitumor Phenotype in GBM.

**Fig. 4.**
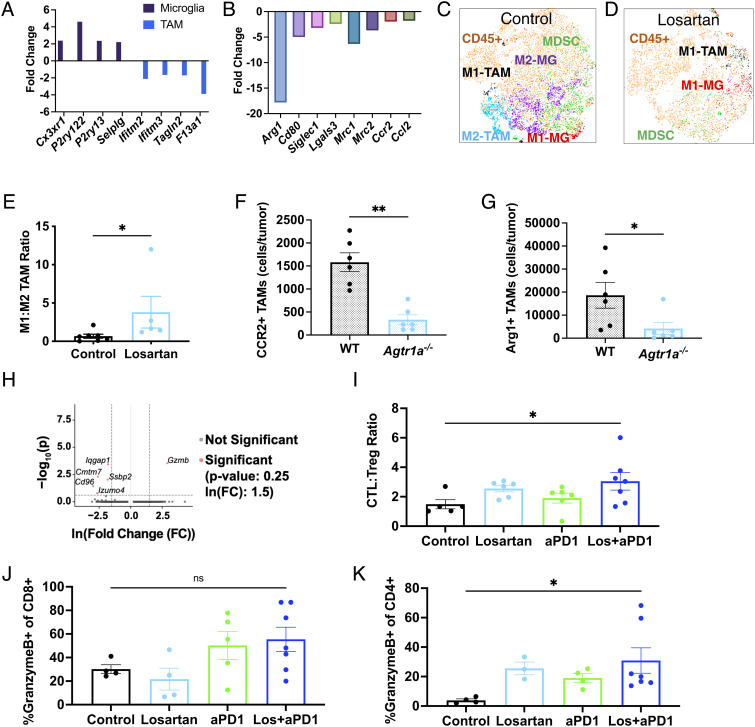
Losartan promotes antitumor immunity in the GBM TME. Applying the human-derived signatures from our previous work (16), losartan is found to enrich microglia-like signatures and downregulate global (*A*) and M2-like (*B*) TAM signatures vs. controls as assessed in bulk RNASeq samples from GL261 (n = 3). t-distributed stochastic neighbor embedding (t-SNE) plots of flow cytometry data of myeloid populations reveal (*C*) a diverse and largely immunosuppressive (“M2”) microenvironment in GL261 controls that is (*D*) reprogrammed by losartan treatment to feature fewer myeloid cells that are polarized for anti-tumor (“M1”) activity (MG, microglia). (*E*) Losartan increases the ratio of anti- to pro-tumor TAMs, assessed via flow cytometry (n = 5 to 7). Highly suppressive TAM subsets (*F*) CCR2+ and (*G*) Arg1+ (of CD45hiCD11b+F4/80+) are downregulated in GL261 tumors implanted in *Agtr1a^−/−^* mice compared to those implanted in wild-type C57Bl/6 mice. (*H*) scRNASeq of CD8^+^ T cells reveals heightened *Gzmb* expression under combined treatment compared to anti-PD1 monotherapy. Losartan+anti-PD1 treatment increases (*I*) cytotoxic (CTL; CD45+CD3+CD8+GranzymeB+) to regulatory (Treg; CD45+CD3+CD4+FoxP3+) T cell ratios in the tumor, and effector Granzyme+ CD8 (*J*, not significant) and CD4 (*K*) T cells in the cervical lymph nodes. (Sequencing: all FDR q-values < 0.25, FC > |2|, adjusted *P* values < 0.05. Flow cytometry: Mann–Whitney unpaired *t* test or one-way ANOVA with Tukey’s post hoc test; *P < 0.05.)

To further explore the beneficial mechanisms of losartan on the TME, we next examined tumor-associated macrophages (TAMs) and resident microglia, as both human and murine GBMs are highly infiltrated by these cells. From bulk RNASeq analyses, we found that losartan upregulated microglia-associated genes (Fig. 4*A*) and reduced the expression of global (Fig. 4*A*) and protumor (“M2-like”) TAM-associated genes (Fig. 4*B*).

Using flow cytometry, we found fewer myeloid cells in losartan-treated tumors with reduced M2-like TAM, microglia, and myeloid-derived suppressor cell compartments (Fig. 4 *C* and *D*) and an increased ratio of anti-/pro-tumor (“M1-like/M2-like”) TAMs (Fig. 4*E*). Moreover, protumor TAM populations were significantly reduced in angiotensin type 1 receptor knockout (*Agtr1a^−/−^*, i.e., the molecular target of losartan*)* mice (Fig. 4 *F* and *G*).

### Losartan Enhances Effector T Cell Function in GBM during ICB Therapy.

Based on the ability of losartan to repolarize the myeloid compartment, we next tested the effects of losartan on T cell function during ICB treatment. We found via scRNASeq that CD8^+^ T cells from losartan+anti-PD1-treated tumors had higher expression of *Gzmb* compared to anti-PD1 monotherapy (Fig. 4*H*). By flow cytometry, we found a significantly increased ratio of cytotoxic Granzyme B+ CD8^+^ T cells to regulatory FoxP3+ CD4^+^ T cells during combined losartan+anti-PD1 treatment (Fig. 4*I*), as well as an increase in the overall percentages of granzyme B+ effector T cells (CD8, Fig. 4*J*, and CD4, Fig. 4*K*) in the draining cervical lymph nodes.

Collectively, our results suggest that losartan can reprogram the GBM TME from immunosuppressive to immunostimulatory. Thus, we next explored the ability of losartan to enhance the survival of tumor-bearing mice with ICB therapy.

### Losartan Enhances ICB Efficacy without or with the Standard of Care (SOC).

**Fig. 5.**
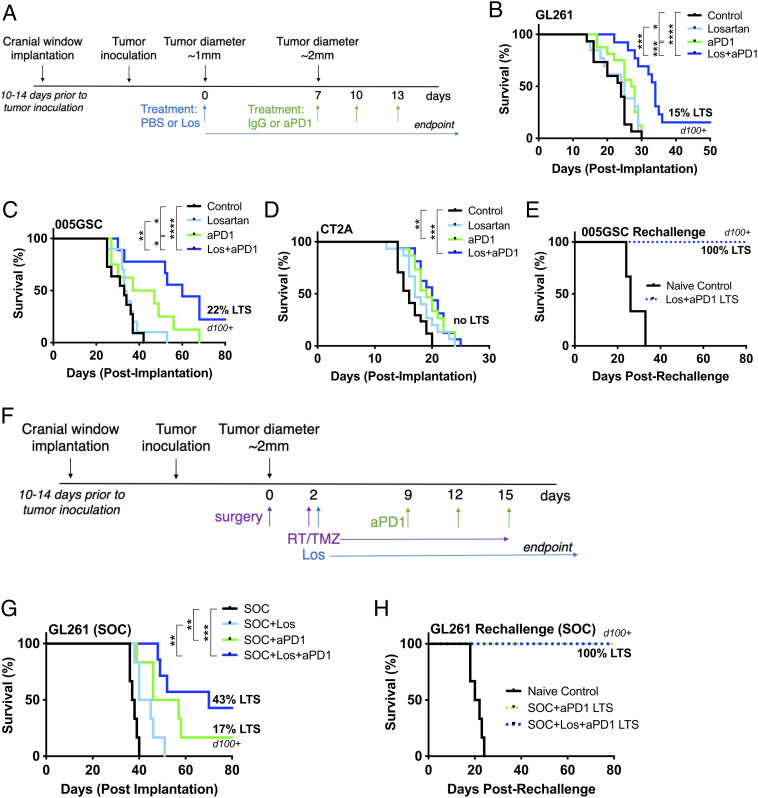
Losartan improves survival under anti-PD1 treatment with and without the SOC. Losartan enhances the survival benefit of anti-PD1 therapy in (*A*) GL261 and (*B*) 005 GSC tumor models with 15% and 22% LTSs, respectively, with no detectable tumors via microultrasound imaging through transparent cranial windows for over 100 d (d100). In addition to lack of increased edema in the face of ICB treatment (Fig. 2*C*), (*C*) the CT2A model displays only a modest response to anti-PD1 therapy that does not result in LTSs nor is improved by the addition of losartan treatment. (*D*) Long-term surviving mice in the 005 GSC model reject a second tumor inoculation, suggesting the formation of an immune memory response. (*E*) The GL261 model subjected to SOC (*F*) therapy shows an improvement (*G*) in response to anti-PD1 (16% LTSs) that is tripled (43% LTSs) in combination with losartan. (*H*) Long-term surviving mice in the GL261 SOC model reject a second tumor rechallenge. (Log-rank Mantel–Cox test; *P < 0.05; **P < 0.01; ***P < 0.001; ****P < 0.0001.)

Based on the beneficial TME effects of losartan, we designed our survival studies to administer losartan 7 d prior to and throughout anti-PD1 treatment (Fig. 5*A*). In GL261 and 005 GSC models, we found that losartan+anti-PD1 antibody doubled animal survival over anti-PD1 monotherapy, and ~20% of the mice survived long-term and rejected subsequent tumor rechallenge (Fig. 5 *B*–*E*). However, in the CT2A model (Fig. 5*D*), we observed only a modest benefit of anti-PD1 therapy; adding losartan failed to further enhance ICB efficacy. This is not unexpected, given that CT2A has higher ECM content (*SI Appendix*, Fig. S4), is refractory to ICB (17), and did not exhibit increased edema under anti-PD1 treatment (Fig. 2*C*).

In GL261 tumors, we found that the SOC treatment (surgical resection, radiation, and temozolomide; Fig. 5*F*) enhanced the anti-PD1 outcome to produce 16% long-term survivors (LTSs) (Fig. 5*G*). Long-term survival almost tripled to 43% when losartan was added to the SOC+anti-PD1, and these surviving mice rejected tumor rechallenge (Fig. 5*H*).

### Immune TME Biomarkers from Bihemispheric Tumor Model Predict Individual Response to Losartan+ICB Therapy.

Because we observed variable responses in individual mice to losartan+anti-PD1 therapy, we sought to identify predictive biomarkers informed by the GBM immune compartment prior to therapy. Building on our recent bilateral breast cancer model (18), we designed a bihemispheric brain tumor model to simultaneously profile immune cells and measure treatment response in individual mice.

**Fig. 6.**
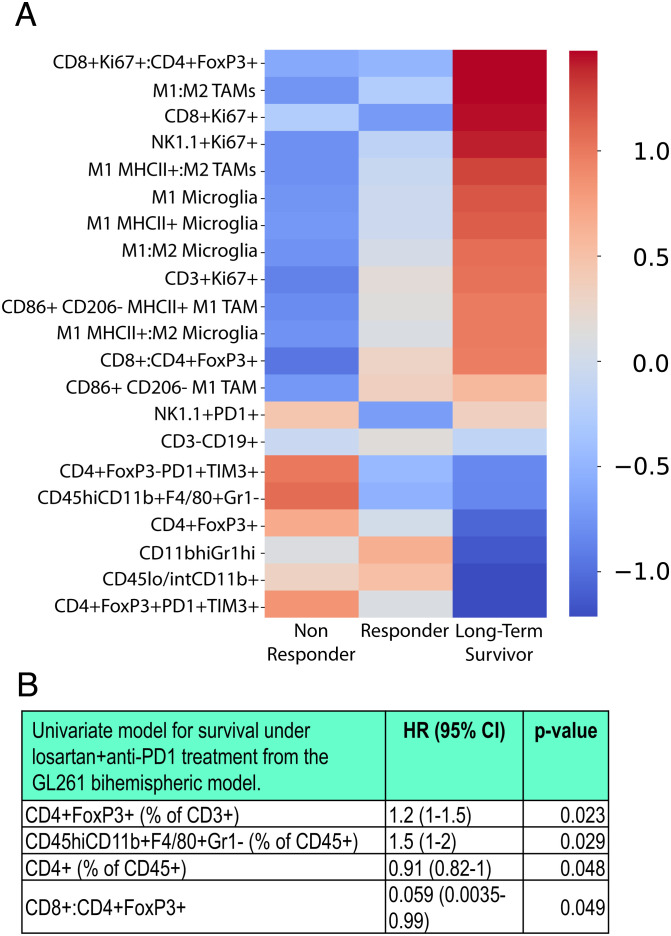
Bihemispheric model reveals predictors of response to losartan+anti-PD1 treatment. The bihemispheric mouse model can be used to resect one tumor for biomarker analysis prior to losartan+anti-PD1 treatment which has variable responses in GL261-bearing mice (n = 9). (*A*) Using flow cytometry, immune cells were profiled in individual mice under combinatorial therapy. As indicated by the heat-map *z*-scores (transformed relative populations of immune cell classes), LTSs have distinguished pretreatment biomarker signatures that indicate that strong antitumor immunity is present in the tumor prior to therapy. (*B*) The presence of CD4 T cells and higher ratios of CD8 to regulatory T cells in the GBM TME before therapy initiation are predictive of improved survival, while the presence of T regulatory cells and TAMs are associated with decreased survival, assessed via proportionate hazard model*s.* (*P* values derived from univariate Cox regression model; HR, hazard ratio; CI, confidence interval.)

Mice were implanted with two identical GL261 tumors in contralateral hemispheres (*SI Appendix*, Fig. S7). We resected one tumor for biomarker analysis prior to the initiation of losartan+anti-PD1 therapy. Each resected tumor was profiled for immune cells using flow cytometry. Each mouse (now bearing its remaining nonresected tumor) was evaluated for individual response to losartan+anti-PD1 therapy. Mice were classified based on survival as nonresponders, responders (improved median survival), and LTSs (no detectable tumor) (*SI Appendix*, Fig. S7). We found that, before the initiation of treatment, tumors from long-term surviving mice had superior antitumor immune profiles compared to nonresponders and responders, including increased ratios of cytotoxic Granzyme B+ CD8 T cells to regulatory FoxP3+ CD4 T cells and “M1-like” to M2-like TAMs and microglia (Fig. 6*A*). Immune biomarkers (T regulatory cells, TAMs, CD4 T cells, and cytotoxic to regulatory T cells ratios) were significantly correlated with survival via univariate Cox proportional hazard models (Fig. 6*B*).

## Discussion

Cerebral edema, a hallmark of GBM, is further exacerbated in a fraction of patients under PD1/PD-L1 treatment (1, 2). We sought to identify an agent that could be used in lieu of immunosuppressive corticosteroids—known to compromise ICB efficacy and effector T cell function (19–21)—to control ICB-induced edema.

Losartan is a small-molecule ARB commonly prescribed as an antihypertensive agent. Losartan can cross the BBB, and ARB use has been reported to be associated with reduced brain edema and lower steroid dosages in GBM patients undergoing chemoradiation treatment (4, 5, 22, 23). However, the steroid-sparing edema control mechanism of ARBs is not fully understood. In two syngeneic GBM models, we showed that losartan prevented anti-PD1-induced edema. Brain edema is attributed largely to overexpression of VEGF, which increases vascular permeability (24, 25). However, bevacizumab—an anti-VEGF antibody that can control edema—failed to improve OS in GBM patients under ICB therapy (1, 21), suggesting a VEGF-independent mechanism for ICB-induced edema.

Our sequencing and CD8^+^ T cell blockade studies indicate the involvement of inflammatory edema. Using scRNASeq analysis, we derived a signature of edema prevention in TECs that included downregulation of MT-MMP-1 and -2 by losartan. T cell interactions with endothelial cells increase MMP expression (8), which can disrupt tight junctions, leading to a compromised BBB (6). However, MT-MMP-1 and -2 have not yet been linked directly to cerebral edema. Our study demonstrates the role of MMPs in mediating anti-PD1-induced edema, generating a working model that CD8^+^ T cells infiltrating into the GBM TME in response to ICB interact with TECs, inducing their increased expression of MT-MMP-1 and -2. This results in a disrupted blood–tumor barrier and increased edema. Importantly, although losartan can reduce VEGF (3), our results indicate that ICB-induced edema is not VEGF-dependent, but rather due to an inflammatory response.

The immunosuppressive nature of the GBM TME stems from multiple factors. Abnormally high ECM deposition is a key contributor; HA and fibrillar collagens are expressed several-fold higher in GBM than in normal brain tissues (26, 27). This contributes to elevated solid stress that impairs perfusion by compressing tumor blood vessels (28). Reduced perfusion limits tumor oxygenation, drug delivery, and trafficking of antitumor immune cells into the GBM TME. This hostile TME contributes to exclusion and exhaustion of cytotoxic T lymphocytes (CTLs) while promoting the infiltration and activation of immunosuppressive Tregs and protumor myeloid cells including TAMs (29, 30). We and others have shown that losartan decreases transforming growth factor beta (TGF-*β*) in mice and cancer patients, thus promoting immune stimulation in non-central nervous system (CNS) tumors (11, 12, 31). However, these effects and the underlying mechanisms have not been investigated in GBM.

Our results indicate that losartan repolarizes TAMs and microglia—both of which promote immunosuppression and are associated with poor prognosis in GBM (32, 33). Losartan has been shown to reduce monocyte recruitment to the TME and suppress tumor growth via inhibition of C-C chemokine receptor type 2 (CCR2) signaling in non-CNS tumors (34, 35). We recently found that high expression of the protumor myeloid receptor CCR2 is associated with poor prognosis in GBM patients and that targeting CCR2 enhances ICB outcome in GBM models (36). Our results here indicate that angiotensin inhibition not only reduces the presence of CCR2-positive TAMs and other pro-tumor myeloid cells but also reprograms the compartment to an anti-tumor phenotype. In extracranial mouse and human tumors, we have linked losartan (and similar ARBs) to antitumor T cell gene expression, presence, and activity (10, 12, 31, 37, 38). Here, we observed improved effector T cell infiltration and function during combined losartan+anti-PD1 therapy. Importantly, although losartan reduces inflammatory responses that contribute to ICB-induced edema, it does not abrogate antitumor immune activity.

We have shown that losartan (and similar ARBs) can improve response to cytotoxic and ICB in pancreatic and metastatic breast cancer mouse models, respectively (10, 11). Here, we found in GBM that losartan improves anti-PD1 outcomes in the 005 GSC and GL261 models but not in CT2A. This could be due in part to excess ECM deposition in CT2A compared to other models, as well as its lack of responsiveness to ICB, and exclusion and exhaustion of CD8 T cells even in the face of anti-PD1 therapy (17, 39). This supposition explains the lack of ICB-induced inflammatory edema in the CT2A model. To lay the groundwork for future clinical translation, we used our recently established SOC model (17) and further improved the durability of losartan+anti-PD1. The lack of secondary tumor formation after rechallenge in “cured” mice suggests the formation of an immune memory response.

Variable patient response to ICB therapy is a stark and challenging clinical reality. There is an unmet need to identify robust and predictive biomarkers of ICB response, due in part to a lack of mechanistic insights into what drives resistance vs. response. This is particularly the case for GBM patients who present with heterogeneous immune landscapes that may drive variable responses to ICB (40–42). Indeed, we observed differential responses within a single treatment arm, even in genetically identical mice bearing tumors grown from the same model and batch of GBM cells.

Building on similar approaches in brain, breast, and subcutaneous sites (18, 43), we developed a bihemispheric tumor model to predict response to losartan+anti-PD1 immunotherapy. Unlike previous studies, however, we utilized this “resection-and-response” approach to evaluate the composition of the GBM immune compartment prior to ICB therapy. Flow cytometry analyses from the bihemispheric model revealed that an immunostimulatory (or “hot”) immune compartment in the TME prior to losartan+anti-PD1 is associated with LTSs. This is in line with a recent retrospective transcriptomic analysis showing that patients with “immune-favorable TMEs” benefit the most from immunotherapy (44). Strikingly, our finding that higher populations of Ki67+CD8+ proliferating CTLs in the treatment-naive murine TME are predictive of long-term survival under losartan+anti-PD1 therapy is directly in line with our recent clinical study where we found increased circulating Ki67+CD8+ CTLs early during durvalumab (anti-PD1) therapy in treatment-naive newly diagnosed GBM patients not on dexamethasone who had better progression-free and OS (21). This approach allows us to establish predictive biomarkers that could be used to inform selection of GBM patients who may respond to losartan+ICB in future clinical trials based on their tumor immune compartment at the time of surgical resection.

A phase III prospective trial with losartan in GBM recently failed to improve median OS in combination with the SOC (45). Similarly, our preclinical results indicate that losartan does not improve OS in GBM mouse models under the SOC unless it is administered in conjunction with ICB. In canine glioma models, losartan was recently found to reprogram the TME and improve objective responses to a tumor-targeting vaccine (46). Retrospective studies [e.g., in nonsmall cell lung, gastrointestinal, and genitourinary cancers (47–49)] suggest that patients under ASIs may have a better response to ICB therapy. Losartan is also under clinical testing for ICB combined with cytotoxic therapy in pancreatic ductal adenocarcinoma patients (NCT03563248) based on our successful phase II trial stemming from our preclinical findings (50). The results of the current study warrant testing combined losartan and ICB therapy in the clinic, along with tissue-based biomarkers identified here for patient selection.

## Materials and Methods

### Patient Cohorts.

A total of 120 patients with pathologically confirmed World Health Organization CNS grade 4 GBM or astrocytoma were identified that were treated with PD1 or PD-L1 ICB at the time of tumor recurrence from December 2013 to November 2020. The analysis was conducted with Dana-Farber Cancer Institute institutional review board approval (protocol 19-360). Informed consent was obtained in writing from each patient involved in this study prior to their enrollment. The outcome of interest was the percentage of maximum edema increase during the first 6 mo following the initiation of ICB. The associations between the outcome and patient clinicopathologic features (including age, sex, KPS (Karnofsky performance score), *IDH* (isocitrate dehydrogenase) mutation status, *MGMT* (O(6)-methylguanine-DNA methyltransferase) promoter methylation status, radiotherapy, bevacizumab, baseline enhancing tumor volume, and baseline edema) were evaluated using univariable and multivariable linear regression. Two-sided *P* values < 0.05 were considered significant. As a secondary analysis, OS was assessed using multivariable Cox regression. OS was measured from the start of ICB treatment to death and otherwise censored at the last follow-up.

### Cell Culture.

Three murine syngeneic cell lines from the C57Bl/6 background were utilized in this study: GL261 (provided by the Frederick National Laboratory, National Cancer Institute), CT2A (provided by Thomas N. Seyfried, Boston College), and 005 GSC (provided by Samuel D. Rabkin, Massachusetts General Hospital). Low-passage parental cell stocks—lacking transfection of potentially immunogenic luciferase or fluorescent reporters—were utilized for all studies with one exception: Green fluorescent protein (GFP)+ GL261 cells were used for the multiphoton microscopy of BBB/BTB (blood-tumor-barrier) permeability (described below under “Intravital Imaging”). All cells were subjected to suspension culture techniques to produce neurospheres and were grown in serum-free conditions using the NeuroCult NS-A proliferation kit (Stemcell Technologies). As described below under “Treatment,” commercially available ICB antibodies (from BioXCell) with an IgG2a isotype were utilized. Thus, in contrast to previous preclinical GBM investigations (51), and in line with recent findings from our group (17)*,* all of the cell lines utilized here are resistant to anti-PD1 monotherapy.

### Animal Models.

#### Mice.

C57Bl/6 and *Agtr1a^−/−^* mice were obtained from the Edwin L. Steele Laboratories, Massachusetts General Hospital. *TCRalpha/beta^−/−^* mice were obtained from Arlene H. Sharpe’s laboratory at the Blavatnik Institute, Harvard Medical School. Male and female mice were used, aged 6 to 8 wk at the start of experiments. Animal protocols were approved by and performed in accordance with the Institutional Animal Care and Use Committees (Massachusetts General Hospital/Harvard Medical School) and the Association for Assessment and Accreditation of Laboratory Animal Care International.

#### Tumor treatment.

Brain tumor implantation in the forebrain (50,000 to 100,000 cells), cranial window surgery, and tumor resection as part of the SOC treatment regimen were conducted as previously described (17, 52, 53). Mice were allowed to recover for 10 to 14 d after cranial window surgery prior to tumor implantation and for 2 d after resection surgery (as part of the SOC or bihemispheric model) prior to treatment initiation.

When tumors reached 1 mm in diameter (7 to 10 d post-implantation), mice were treated daily with phosphate-buffered saline (PBS;control) or losartan (Selleckchem) daily at 60 mg/kg until study endpoint. After 1 wk of losartan pretreatment, mice were treated with IgG (control) or anti-PD1 (BioxCell, RMP1-14) every 3 d for three doses at 200 μg/mouse. SOC mice received concurrently with losartan: 5 d of consecutive radiotherapy (2 Gy/d) and 10 d of consecutive chemotherapy (temozolomide, Selleckchem) at 25 mg/kg. All drugs were injected *i.p*. For flow cytometry, scRNASeq, intravital imaging, histology, and edema measurements, mice were imaged/sacrificed after the third dose of anti-PD1 and/or 2 wk of losartan treatment.

#### Bihemispheric model.

Here, 10 to 14 d after cranial window surgery, mice are implanted with two identical tumors from the same batch of cells, one in each forebrain hemisphere. Tumor development is monitored via 3D-microultrasound; when each tumor reaches 2 mm diameter, one tumor is surgically excised. Each excised tumor is subjected to biomarker analysis (in this study, immune profiling of the GBM TME) prior to treatment initiation. Two days after surgical resection, each mouse bearing its remaining tumor undergoes concurrent losartan+anti-PD1 therapy. At endpoint, mice are classified as nonresponders, responders (improved median survival), and LTSs (no detectable tumor) and evaluated for predictive biomarkers from the resected pretreatment tumor. The heatmap of immune cell populations or their ratios (z-score transformed) for each survival classification was generated using the Seaborn 0.9.0 package in the Python language environment. Relative pop The Cox proportional hazard regression models were generated using the “survminer” and “survival” packages in the R platform.

#### Flow cytometry.

Single-cell suspensions were prepared from tumors and cervical draining lymph nodes that were isolated and dissected under a stereotactic microscope. Cells were stained and processed (on a BD LSRFortessa X-20 Cell analyzer) and analyzed (FlowJo, Tree Star) as previously described (17). The following antibodies from BD Biosciences, EBioscience, and BioLegend were used at 1:200 dilutions: CD45-BV605, CD3-BV785, CD4-BV640, CD8-BV711A, NK1.1-APC; FoxP3-BV421; PD1-PerCP710A; TIGIT-PE Cy7; TIM3-PE; CD19-BV510; KI67-FITC; GranzymeB-PE Cy7; CD11b-BV785; MHCII-BV605; F4/80-PerCP Cy5.5; CX3CR1-APC; CD206-PE CY7; CD86-BV650; CCR2-PE; and GR1-AF700.

#### Edema measurements.

Edema in the tumor was assessed immediately after animal sacrifice via wet/dry weight analysis to determine the water content as previously described (53).

#### Histology and immunostaining.

Brains with tumors were prepped and stained for histology as previously described (52) and imaged on a TissueFAXS (TissueGnostics) slide scanner at the Ragon Institute, Massachusetts Institute of Technology.

### Intravital Imaging.

#### 3D microultrasound.

Tumor size was visualized by 3D microultrasound in anesthetized mice through the transparent cranial windows (52). Ultrasound was also used to measure tumor deformation as a readout of solid stress, following previous methods (14).

#### Multiphoton analysis of BBB/BTB permeability.

Multiphoton images were acquired in anesthetized mice through transparent cranial windows using a custom-built multiphoton microscope coupled to a mode-locked femtosecond pulsed Ti:Sapphire laser with a Zeiss 25 × 1.05 NA water dipping objective. The 820-nm multiphoton laser excited fluorescein and TAMRA and the emission were collected using 535- to 578-nm and 610- to 685-nm bandpass filters, respectively. Retroorbital injection of TAMRA-conjugated bovine serum albumin (67 kDa, Invitrogen, 0.1 mL of 10 mg/mL) was performed. In vivo images were acquired 60 min after TAMRA injection. All images were subjected to threshold processing, and the extravascular fluorescent intensity was measured using the integrated density measurement function (in ImageJ).

#### OCT imaging of tumor perfusion.

In vivo imaging of perfused vessels was achieved via a custom-built OCT system as previously described (15). Mice were anesthetized and imaged throughout losartan treatment. A depth-resolved profile was generated each day, and the raw tomograms were processed as previously described. Images across multiple days were coregistered using the scale-invariant feature transform algorithm in ImageJ and Python.

### scRNASeq.

#### Processing of murine GBM samples for scRNASeq.

Following the single-cell suspension techniques of flow cytometry, tumor cells were blocked in 1% bovine serum albumin in phosphate-buffered saline solution (1% BSA/PBS). Cell suspensions were subsequently stained for flow cytometry for 30 min at 4 °C using antibodies specific for CD45 [30F11]-VioBlue from Miltenyi, CD3 [145-2C11]-PE from Biolegends, and CD31 [MEC 13.3]-PE from BD Biosciences. Cells were washed with cold PBS and then incubated for 15 min in 1.5 mL of 1% BSA/PBS containing 1 μM calcein AM (Life Technologies) and 0.33 μM TO-PRO-3 iodide (Life Technologies). Sorting was performed with the FACS Aria Fusion Special Order System (Becton Dickinson) using 488-nm (calcein AM, 530/30 filter; CD3-PE, 585/42 filter), 640-nm (TO-PRO-3, 670/14 filter), and 405-nm (CD45-VioBlue, 450/50 filter) lasers. Standard, strict forward scatter height vs. area criteria were used to discriminate doublets and gate-only singleton cells. Viable cells were identified by staining positive with calcein AM but negative for TO-PRO-3. We sorted individual, viable, CD45^+^CD3^−^ and CD45^+^CD3^+^ immune, and CD45^−^ nonimmune single cells into 96-well plates containing cold TCL buffer (QIAGEN) with 1% beta-mercaptoethanol. Plates were frozen on dry ice immediately after sorting and stored at −80 °C prior to whole transcriptome amplification, library preparation, and sequencing.

#### Preparation of scRNASeq libraries.

Smart-seq2 whole transcriptome amplification, library construction, and sequencing for malignant cells and microglia were performed as previously published (16, 54). Single-cell cDNA and sequencing libraries for T cells and TECs were prepared using the SMART-seq2 protocol with multiple adaptations (20): During the dT annealing step, trehalose (1M) was used instead of water to make up the reaction volume. For the reverse transcription step, Maxima RNaseH-minus RT (200 U/mL) was added at 2 U/mL, water was replaced with trehalose (1M), and betaine was omitted from the reaction. RT was performed at 50 °C for 90 min followed by 85 °C for 5 min. PCR preamplification was performed for 21 cycles for T cells and for 22 cycles for endothelial cells.

#### scRNASeq data processing.

Sequencing data were processed from raw reads to gene expression matrices, starting with fbcl2fastq (v2.20.0) to generate demultiplexed FASTQ files. Bowtie was used to align the resulting paired-end scRNASeq reads to the mouse transcriptome (mm10) (55). Gene expression levels were quantified as transcripts-per-million (TPM) by running RSEM (v1.2.19) in paired-end mode. Gene expression levels were quantified as TPM by running RSEM (v1.2.19) in paired-end mode. Total transcripts per cell were normalized to 100,000 (TP100K), as the estimated the complexity of single-cell libraries prepared by SMART-Seq2 (54). The values were then log-transformed to report gene expression as E = ln(TP100K + 1).

#### Quality control of scRNASeq.

A gene was considered to be detected in a given cell if its TP100K was greater than 0. Cells with either less than 1,000 or greater than 8,000 unique genes detected were excluded; or if a cell had fewer than 20 housekeeping genes, based on a previously identified gene set (54), it was excluded.

#### Cell type and cell state identification.

Following the methods of our recent study (20), cell-type states were identified using the R package Seurat (v4.0.0) (56). Genes identified as highly variable were selected for downstream clustering using FindVariableGenes. The following thresholds were used for the mean expression (x) and the variance to mean ratio (y): x.low.cutoff = 0.1, x.high.cutoff = 7, y.cutoff = 0.5. The 1,500 variable genes that were most commonly shared across all samples were selected. Next, gene expression of each gene was centered around a zero mean using ScaleData. Principal components analysis (PCA) was performed with RunPCA, and Louvain clustering was performed on the top 20 principal components (PCs) using FindClusters, with the resolution parameter set to 0.4 and k for the k-nearest neighbor algorithm set to 30. Each cluster of cells was analyzed for differentially expressed genes using the *t* test implemented in FindMarkers while adjusting *P* values for multiple hypothesis testing via Bonferroni post hoc tests. Uniform Manifold Approximation and Projection (UMAP) embedding of the top 20 PCs (using RunUMAP with the following settings: min_dist = 0.5, number of neighbors = 30, and distance metric = Euclidean) was used to visualize clustering results, followed by cell-type annotation.

#### Inference of copy number alterations.

Default parameters of inferCNV were used to confirm annotation of malignant cell clusters, as implemented in the R code https://github.com/broadinstitute/infercnv (57). The clusters annotated as T cells, endothelial cells, myeloid, microglia, B cells, NK cells, and oligodendrocytes were used as reference. A subset of the nonmalignant cells were then used as a reference; no copy number alterations (CNAs) were detected in the nonmalignant cells that were not provided as a reference.

CNAs were scored by first defining the overall CNA level of a given cell as the sum of the absolute CNA estimates across all genomic windows. Cells were then identified with the highest overall (top 10%) CNA level and the average CNA profile of these cells was considered as the CNA profile of the sample. Next, the CNA-R-score was computed for each cell using the Spearman correlation coefficient obtained by comparing its CNA profile to the inferred CNA profile of the sample. Cells with a high CNA-R-score (defined as greater than 25%) were considered malignant by the CNA criterion.

#### Differential gene expression between treatments.

To explore variability between the expression profiles of cell types given a specific treatment, the FindMarkers function was used to identify differentially expressed genes between cells of two treatments of a given cell type. Volcano plots were generated using the R package EnhancedVolcano (v1.13.2) (https://github.com/kevinblighe/EnhancedVolcano). Genes were considered significant with a corrected *P* value < 0.25 and log_2_FC > 1.5.

#### Edema signature score.

The level of edema signature score was calculated using *AddModuleScore*, which calculates the average expression levels of genes in a signature and subtracts from them the average expression levels of control gene sets (54), to examine gene expression signatures within individual cells. The control gene sets were selected to have comparable expression values to the genes in the signature. All genes were placed into 25 bins based on their average expression across all cells. For each gene in a signature, a random set of 10 genes from the same average expression bin as that gene were chosen. This methodology controls for the differences in cell quality and library complexity across single cells.

### Patient Perfusion MRI Data.

Perfusion MRI (pMRI) data were collected from patients from trial NCT00662506 and analyzed using the previously established vessel architectural imaging technique (58). Briefly, image voxels can be distinguished as arterial or venous-dominated. “Tissue function” parameters are shown that are the ratio for mean blood volume and perfusion values corrected for corresponding levels of normal brain tissue. These values are were quantified only from patients with sufficient pMRI quality data. Kaplan–Meier survival comparisons were calculated from the entire dataset of patients on ASIs like losartan vs. those not (non-ASI).

### Statistical Analysis.

Statistics were performed using Prism (GraphPad Software Inc.). Figure legends depict the number of mice used in each experiment (n), the statistical test used, and the visualization (e.g., mean with error bars showing SEM). Differences with *P* < 0.05 are considered statistically significant. Patient data were analyzed via Stata (SEv17.0, StataCorp).

## Supplementary Material

Appendix 01 (PDF)

Dataset S01 (XLSX)

Dataset S02 (XLSX)

Dataset S03 (XLSX)

Movie S1. **Perfused vessels during GBM growth under control (PBS) treatment.** Longitudinal high-resolution angiography in tumor-bearing mice with circular transparent cranial windows (8 mm in diameter) is achieved using Doppler optical coherence tomography (OCT) (15). A GL261 tumor has been implanted in the upper left-hand quadrant of the image. Day 1 represents treatment start date (PBS control) when tumors are randomized at 1 mm in diameter. Untreated tumors feature highly chaotic, irregular and poorly functional vessels that worsen over time.

Movie S2. **Perfused vessels during GBM growth under losartan treatment.** GL261 tumor under losartan treatment features more straightened (“normalized”) vessels with increased overall perfusion compared to control, with maximum benefit to vessel structure and function observed within the first few days of treatment. However, over time, vessels become abnormal again. This is consistent with the transient “window of normalization” that is observed under anti-VEGF therapy (45, 46). In this case it is posited to be due either to insufficient counteraction of VEGF signaling by losartan, and/or compensatory pro-angiogenic mechanisms by the tumor.

## Data Availability

Data are available in the supplemental data (Datasets S1–S3) or by request. All study data are included in the article and/or SI Appendix.
