# Supplementary material for: Losartan controls immune checkpoint blocker-induced edema and improves survival in glioblastoma mouse models
Source: Proc Natl Acad Sci U S A. 2023 Feb 1;120(6):e2219199120. doi: 10.1073/pnas.2219199120 (PMC9963691; doi:10.1073/pnas.2219199120)
Supplement: Supplementary file 1 — Appendix 01 (PDF) [file pnas.2219199120.sapp.pdf]

PNAS

Supporting Information for

Losartan controls immune checkpoint blocker-induced edema and improves survival in glioblastoma mouse models

Meenal Datta*, Sampurna Chatterjee, Elizabeth M. Perez, Simon Gritsch, Sylvie Roberge, Mark Duquette, Ivy X. Chen, Kamila Naxerova, Ashwin S. Kumar, Mitrajit Ghosh, Kyrre E. Emblem, Mei R. Ng, William W. Ho, Pragya Kumar, Shanmugarajan Krishnan, Xinyue Dong, Maria C. Speranza, Martha R. Neagu, J. Bryan Iorgulescu, Raymond Y. Huang, Gilbert Youssef, David A. Reardon, Arlene H. Sharpe, Gordon J. Freeman, Mario L. Suvà*, Lei Xu*, and Rakesh K. Jain*

* Corresponding authors: mdatta@nd.edu, suva.mario@mgh.harvard.edu, lei@steele.mgh.harvard.edu, jain@steele.mgh.harvard.edu

This PDF file includes:

Figures S1 to S7
Tables S1 to S2
Legends for Movies S1 to S2
Legends for Datasets S1 to S3
SI References

Other supporting materials for this manuscript include the following:

Movies S1 to S2
Datasets S1 to S3

Fig. S1.

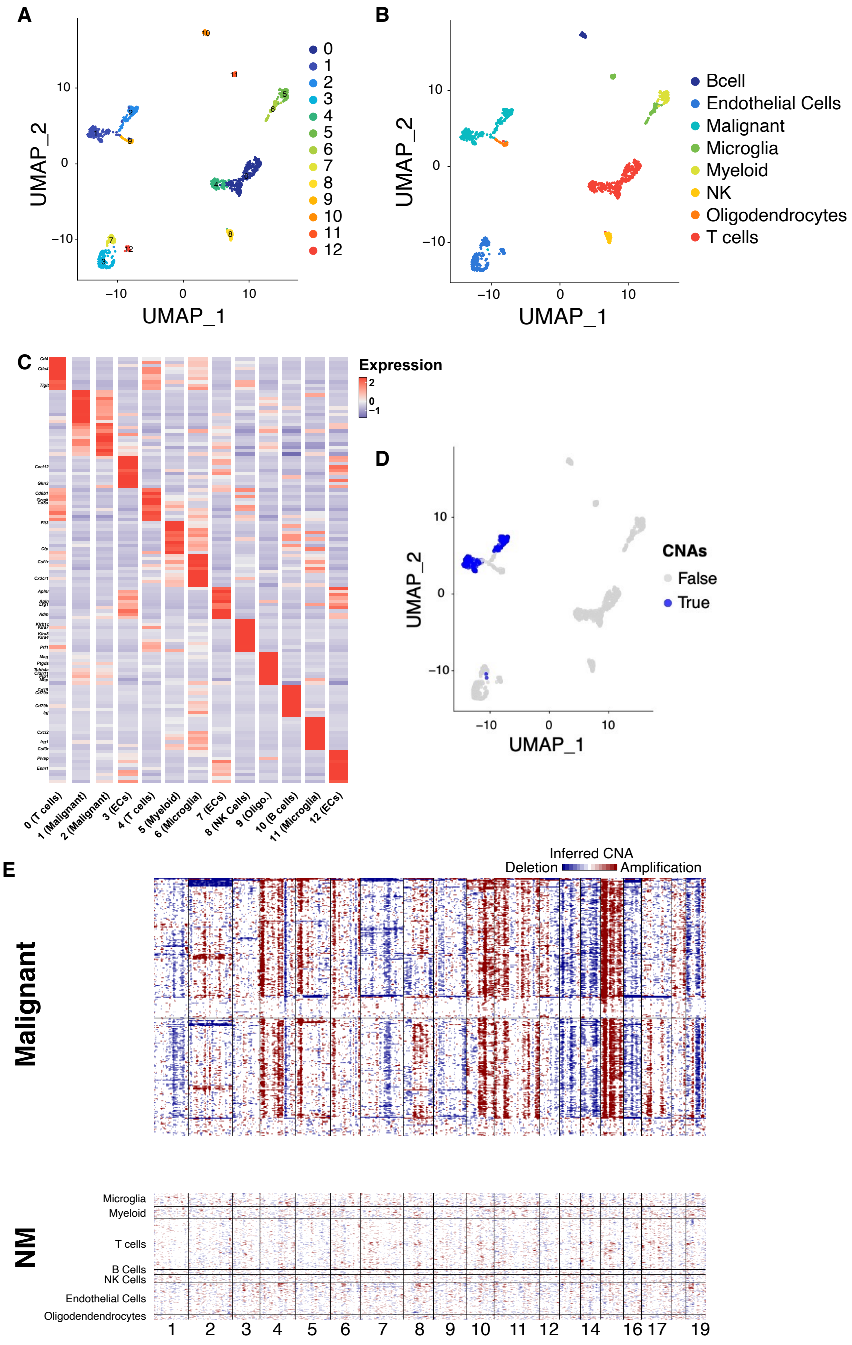

Figure S1. Single-cell RNA sequencing (scRNASeq) reveals a diverse murine GBM TME. ScRNASeq profiling of the GL261 GBM TME resulted in (**A**) uniform manifold approximation and projection (UMAP) visualization of Louvain clustering of all cells identified 12 cell clusters corresponding to (**B**) 8 distinct cell types by differentially expressed genes (Methods; centered, units of ln(TP100k +1)), including tumor endothelial cells (TECs). (**C-E**) Copy number alterations as detected by inferCNV (Methods) with differential gene expression (**B**, **Table S1**) are used to distinguish and annotate (**E**) malignant and non-malignant clusters.

Fig. S2.

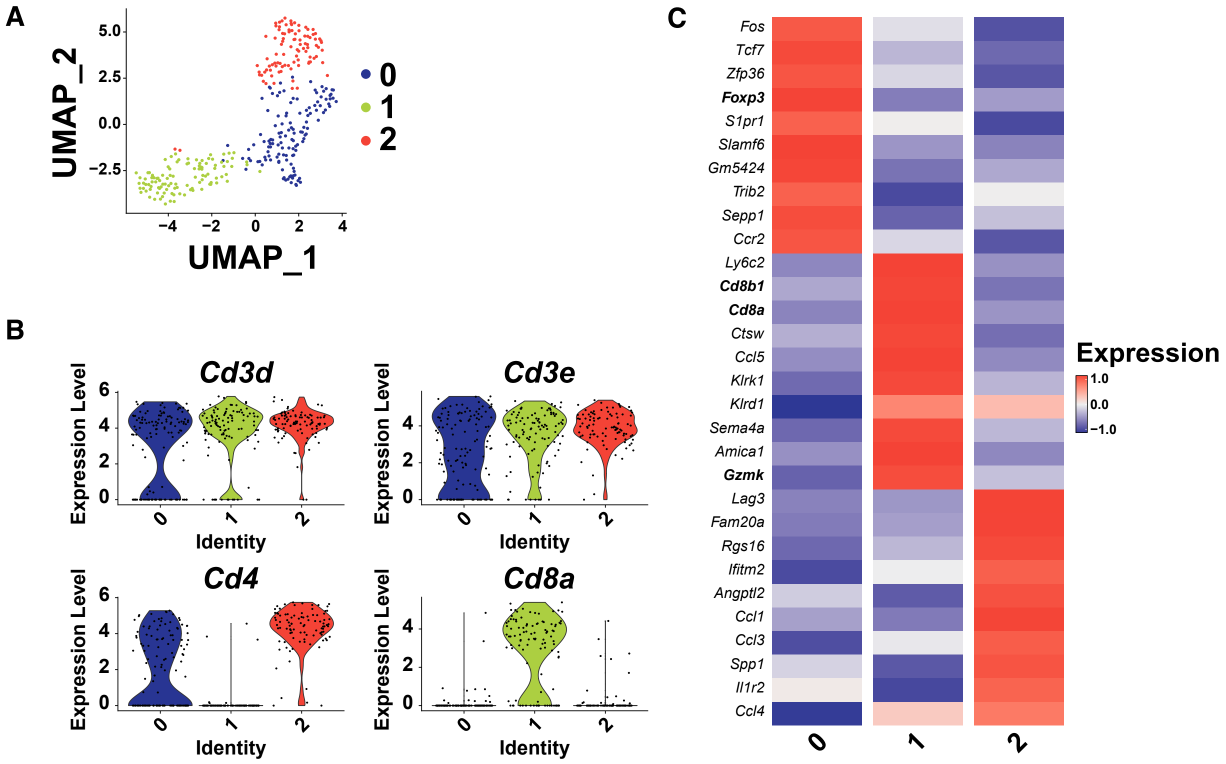

Figure S2. Murine GBM T cells recapitulate those found in human GBM. (**A**) UMAP visualization of Louvain clustering (Methods) of T cells (*Cd3d*+ and *Cd3e*+) identified three clusters of T cells. (**B**) Clusters 0 and 2 are CD4+ T cells and cluster 1 is a CD8+ T cell population. (**C**) Heatmap of differentially expressed genes (centered, units of ln(TP100k +1)) shows Cluster 0 is enriched for T regulatory cell (Treg) markers (*Foxp3* and *Ctla4*), and cluster 1 is enriched for genes such as *Cd8a* and *Gzmk*, a marker of cytotoxicity. These results mimic in part the T cell phenotypes we recently characterized from scRNASeq of human GBM (1).

Fig. S3.

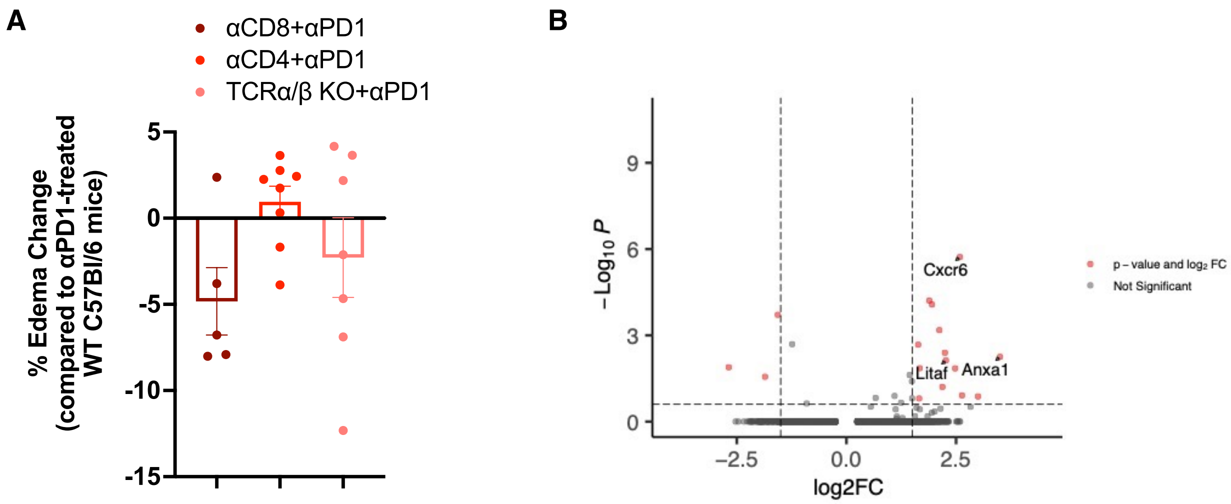

Figure S3. CD8 T cells are important mediators of ICB-induced edema. To identify which infiltrating immune cells may contribute to ICB-induced edema, we used T cell depleting antibodies in wildtype mice bearing GBM, or grew tumors in TCRα/β knockout mice. (**A**) Anti-PD1-induced edema in GL261 GBM model is significantly decreased in mice treated with an anti-mouse CD8 depleting antibody or in TCRα/β knockout mice bearing GL261 tumors, but is not significantly affected by an anti-CD4 depleting antibody (as compared to anti-PD1 treated wildtype C57Bl/6 mice; n=5-8). (**B**) Volcano plot (x-axis: ln(Fold Change); y-axis: -log10(Benjamini Hochberg corrected-p value)) of scRNASeq reveals upregulation of genes linked to lymphocyte-endothelial interactions (e.g., *Cxcr6, Anxa1, Litaf*) in CD8 T cells from anti-PD1-treated tumors vs IgG-treated control tumors.

Fig. S4.

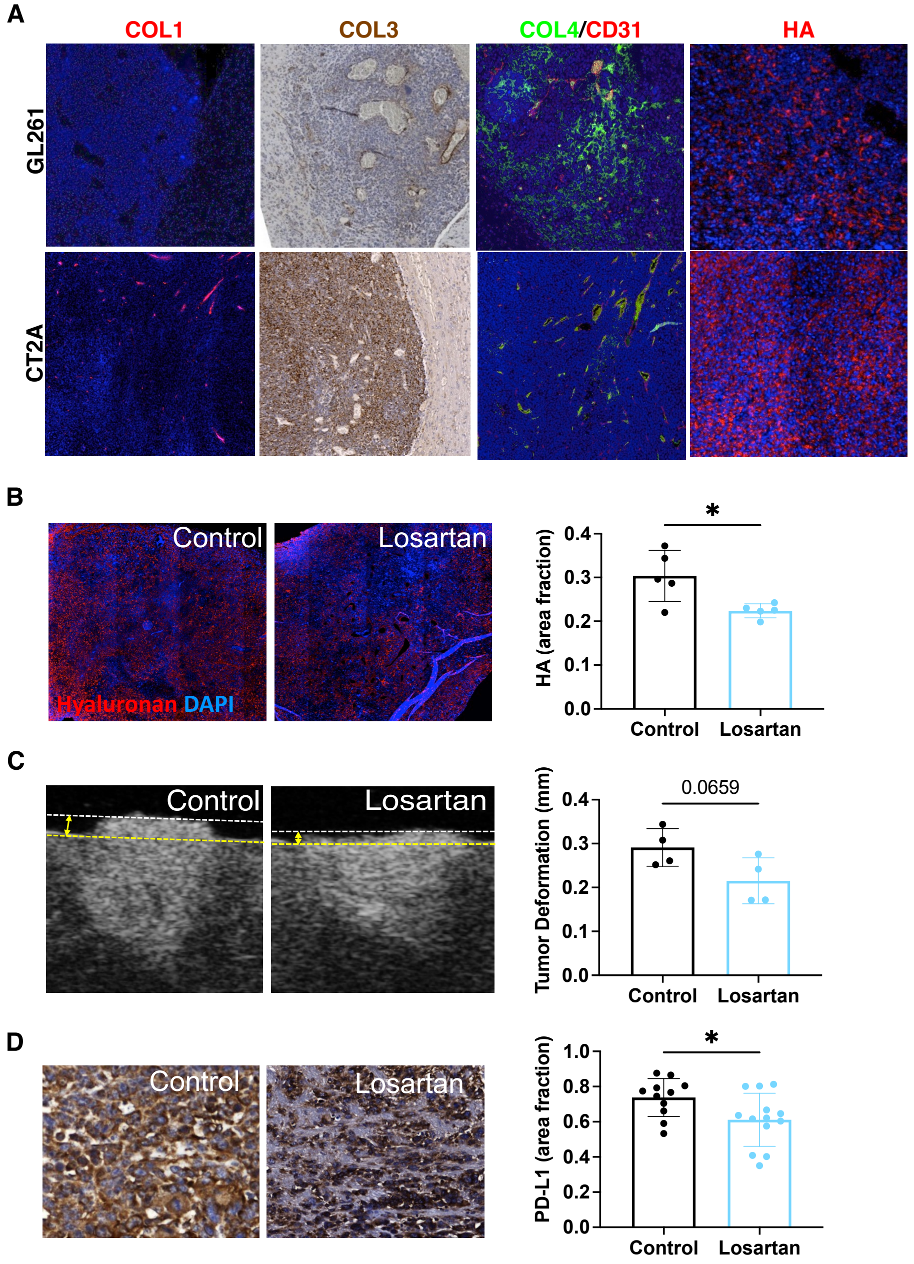

Figure S4. Losartan reduces HA, solid stress, and PD-L1 expression. (A) CT2A tumors express higher amounts of ECM proteins than GL261 including fibrillar collagens (collagens 1 and 3) and HA. In the GL261 model, 2 weeks of losartan treatment reduces (**B**) HA levels as measured and quantified via immunofluorescent imaging (n=5), (**C**) solid stress as measured via 3D microultrasound of tissue (white line) deformation compared to the normal brain (yellow line) reference plane (2) (n=4), and (**D**) PD-L1 protein expression via immunohistochemistry (n=11-13). (*Positive stains quantified as area fractions of total tumor area; Bar plots: mean±SEM; Student's unpaired t-test; * = p<0.05.*)

Fig. S5.

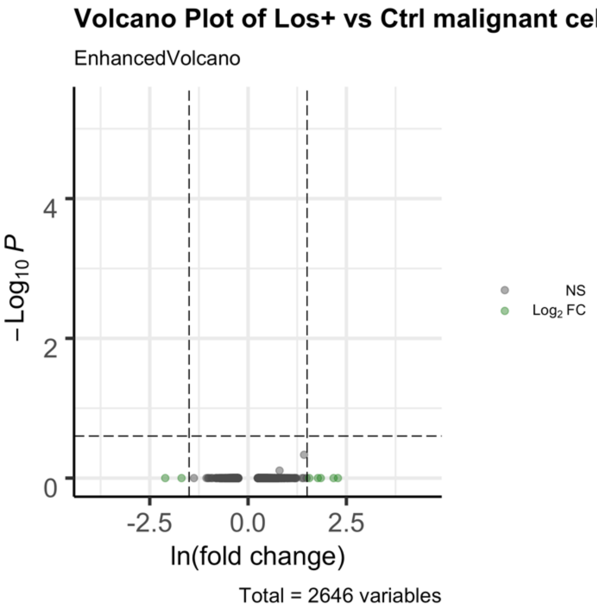

Figure S5. Losartan does not significantly alter GBM cancer cells. Losartan does not exert cytotoxic nor any other notable effects on malignant GL261 cells. The Volcano plot (x-axis: ln(Fold Change); y-axis: -log10(Benjamini Hochberg corrected-p value)) of scRNASeq reveals no statistically significant differentially expressed genes in cancer cells from losartan-treated tumors control tumors.

Fig. S6.

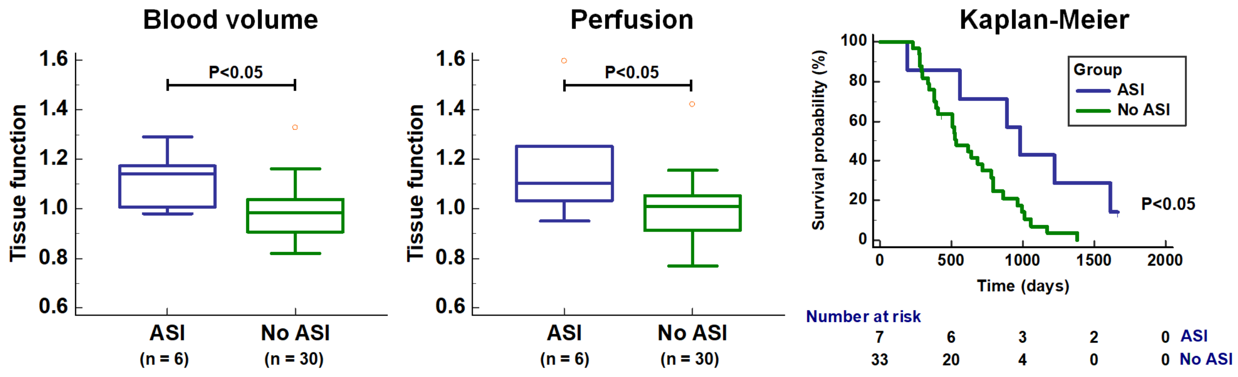

Figure S6. Angiotensin system inhibition usage in treatment-naïve GBM patients is associated with better perfusion and survival. At baseline prior to therapy, perfusion MR image analysis (pMRI) reveals that patients on angiotensin system inhibitors (ASI, n=6 pMRI) like losartan have higher overall blood volume and tumor perfusion than patients not on anti-hypertensives (no ASI, n=30 pMRI). When combined with cediranib, patients on ASI (n=7) have higher overall survival (980 vs 527 days) compared to those not on ASIs (n=33). (*Tissue function: non-dimensional relationship between perfusion profiles of tumor vs normal tissue for venous and arterial areas, as quantified by vessel architectural imaging (3), which was performed as part of the protocol of clinical trial NCT00662506. These values are shown only for patients with sufficient pMRI quality data. Box plots: mean±SEM; Student's unpaired t-test; * = p<0.05; Survival: Log-rank Mantel-Cox test.*)

Fig. S7.

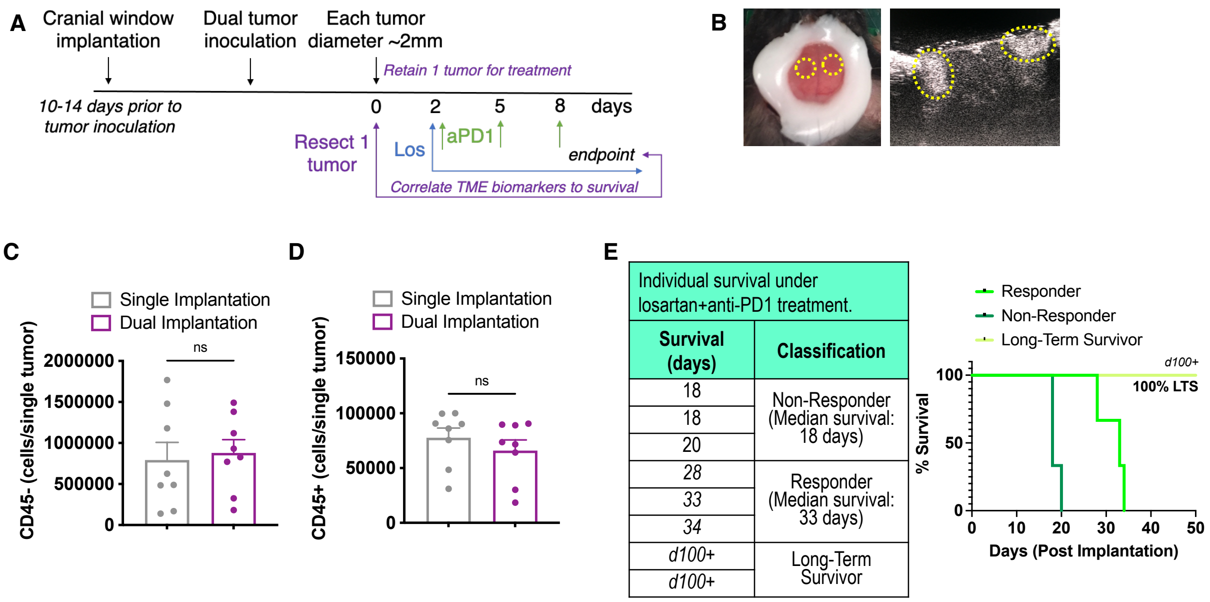

Figure S7. Bihemispheric model predicts individual response to immunotherapy. (**A**) Identical GBM cells are implanted in each hemisphere under transparent cranial windows (for imaging, **B**). After initial tumor growth and prior to therapy, one tumor is surgically resected and subjected to TME analysis. Flow cytometry analysis of the resected tumor shows that the number and proportion of non-immune (**C**, CD45-) vs. immune cells (**D**, CD45+) in a single resected tumor does not vary significantly whether a single or dual implantation has been conducted (n=8). (**E**) Mice bearing the remaining second tumor receive systemic combinatorial immunotherapy (losartan+anti-PD1 treatment) and are evaluated for individual and variable survival responses (n=8). They are classified based on survival as non-responders (median survival similar to control mice), responders (mice with improved median survival that eventually succumb), and long-term survivors (mice with non-detectable tumors). (*Bar plots: mean±SEM; Student's unpaired t-test;* * = *$p<0.05$.*)

Table S1. Clinical factors associated with ICB-induced edema in GBM patients.

	Univariable linear regression for max % change in edema within 6 mos of starting ICB			Multivariable linear regression for max % change in edema within 6 mos of starting ICB		
	Coefficient	(95%CI)	p value	Coefficient	(95%CI)	p value
Age, yr	-1.18/yr	(-5.08-2.71)	0.55	-1.10/yr	(-5.01-2.8)	0.58
Sex						
Female	ref			ref		
Male	39.70	(-40.11-119.51)	0.33	30.54	(-42.12-103.19)	0.41
KPS						
<90	ref			ref		
>=90	46.82	(-31.36-125)	0.24	30.49	(-44.08-105.07)	0.42
n/a	-81.97	(-509.04-345.11)	0.71	-251.05	(-646.4-144.31)	0.21
IDH status						
Wildtype	ref			ref		
Mutant	-88.29	(-337.38-160.8)	0.48	-79.94	(-325.48-165.6)	0.52
n/a	8.77	(-295.01-312.55)	0.96	-70.10	(-373.57-233.38)	0.65
MGMT promoter						
Unmethylated	ref			ref		
Methylated	47.18	(-33.39-127.75)	0.25	41.92	(-33.08-116.92)	0.27
n/a	97.98	(-47.45-243.41)	0.19	126.69	(-24.17-277.54)	0.10
Radiotherapy						
No	ref			ref		
Yes	131.04	(41.26-220.82)	0.005	130.63	(42.47-218.8)	0.004
Bevacizumab						
No	ref			ref		
Yes	-121.39	(-197.16--45.63)	0.002	-92.65	(-167.85--17.45)	0.02
Baseline Enhancing Volume, mm^3	-2.60E-03/ mm^3	(-0.005--0.0002)	0.04	1.68E-03/ mm^3	(-0.0011-0.0044)	0.23
Baseline Edema, mm^3	-1.58E-03/ mm^3	(-0.0022--0.0009)	<0.001	-1.39E-03/ mm^3	(-0.0022--0.0006)	<0.001

Table S2. Multivariable Cox regression analysis of overall survival[1] among glioblastoma patients that received anti-PD-(L)1.

	HR	95%CI	p value
Age, yr	1.04/yr	(1.01-1.07)	0.002
Sex			
Female	ref		
Male	0.90	(0.58-1.39)	0.62
KPS			
<90	ref		
>=90	0.98	(0.63-1.52)	0.93
n/a	0.84	(0.1-7.3)	0.88
IDH status			
Wildtype	ref		
Mutant	1.71	(0.48-6.1)	0.41
n/a	1.46	(0.26-8.19)	0.67
MGMT promoter			
Unmethylated	ref		
Methylated	0.79	(0.5-1.24)	0.31
n/a	1.35	(0.49-3.72)	0.56
Radiotherapy			
No	ref		
Yes	1.70	(0.97-2.99)	0.07
Bevacizumab			
No	ref		
Yes	1.89	(1.2-2.99)	0.006
Baseline enhancing volume, mm^3	1.00004/mm^3	(1.00002-1.00006)	<0.001
Baseline edema, mm^3	1.00000/mm^3	(0.99999-1)	0.15
Maximum % change in edema within 6 months of starting ICB	1.00012/%	(0.99882-1.00143)	0.85

[1]Overall survival was measured from initiation of anti-PD-(L)1 therapy until death, with censoring at last follow-up. (Patient n=120, of which 104 died. HR = hazard ratio, CI = confidence interval.)

Movie S1. Perfused vessels during GBM growth under control (PBS) treatment. (separate file)

Longitudinal high-resolution angiography in tumor-bearing mice with circular transparent cranial windows (8 mm in diameter) is achieved using Doppler optical coherence tomography (OCT) (*15*). A GL261 tumor has been implanted in the upper left-hand quadrant of the image. Day 1 represents treatment start date (PBS control) when tumors are randomized at 1 mm in diameter. Untreated tumors feature highly chaotic, irregular and poorly functional vessels that worsen over time.

Movie S2. Perfused vessels during GBM growth under losartan treatment. (separate file)

GL261 tumor under losartan treatment features more straightened ("normalized") vessels with increased overall perfusion compared to control, with maximum benefit to vessel structure and function observed within the first few days of treatment. However, over time, vessels become abnormal again. This is consistent with the transient "window of normalization" that is observed under anti-VEGF therapy (*45, 46*). In this case it is posited to be due either to insufficient counteraction of VEGF signaling by losartan, and/or compensatory pro-angiogenic mechanisms by the tumor.

Dataset S1. Differentially expressed genes from scRNASeq of GL261 tumors. (separate file)

Top differentially expressed genes for cells from GL261 tumors and resulting cell type subclusters.

Dataset S2. Differentially expressed genes from scRNASeq of TECs from GL261 tumors under losartan and/or anti-PD1 therapy. (separate file)

Top differentially expressed genes for TECs from GL261 tumors for each treatment comparison (sheets 1-6).

Dataset S3. Differentially expressed genes from scRNASeq of CD8+ T cells from GL261 tumors under losartan and/or anti-PD1 therapy. (separate file)

Top differentially expressed genes defining T cell subclusters (sheet 1) from GL261 tumors, as well as treatment comparisons for CD8 T cells ("Cluster 1"; sheets 2-7).

SI References

1. N. D. Mathewson *et al.*, Inhibitory CD161 receptor identified in glioma-infiltrating T cells by single-cell analysis. *Cell* **184**, 1281-1298 e1226 (2021).
2. H. T. Nia *et al.*, Quantifying solid stress and elastic energy from excised or in situ tumors. *Nat Protoc* **13**, 1091-1105 (2018).
3. K. E. Emblem *et al.*, Vessel architectural imaging identifies cancer patient responders to anti-angiogenic therapy. *Nat Med* **19**, 1178-1183 (2013).
